# Management of Painful Vertebral Compression Fracture With Kyphoplasty in a Sever Cardio-Respiratory Compromised Patient

**DOI:** 10.5812/aapm.5030

**Published:** 2012-07-10

**Authors:** Farnad Imani, Helen Gharaei, Poupak Rahimzadeh, Zahra Saffarian

**Affiliations:** 1 Department of Anesthesiology and Pain Medicine, Rasoul-Akram Medical Center, Iran University of Medical Sciences (IUMS), Tehran, Iran

**Keywords:** Kyphoplasty, Fractures, Compression, Osteoporosis

## Abstract

**Introduction:**

Vertebral body compression fractures due to osteoporosis, often lead to pain and disability which can be successfully treated by injecting cement into the vertebral body, a procedure known as Balloon Kyphoplasty. In this procedure, an inflatable balloon is used to restore vertebral body height before injection of the cement. Vertebral compression fractures have been treated conservatively with the bed rest, pain medications, and back bracing to decrease the patient’s pain, but the spine was left in its deformed state. Open surgical treatment can address the deformity, but it is usually reserved for patients with a neurological deficit. Kyphoplasty have been developed as an alternative to surgery for the treatment of painful vertebral compression fractures.

**Case Presentation:**

A 65 year-old female had a compression fracture and decrease height of L1 due to falling down. She had a local severe pain (VAS 8) and tenderness over L1 to L4 vertebra and in her physical examination, there were not any neurologic deficits or bowel and bladder dysfunction. She had a severe cardiovascular disease with low ejection fraction (30%) and had a pace maker, hypertension, diabetes, Chronic obstructive pulmonary disease (COPD), and used about 15 drugs daily. The risk of anesthesia and open surgery was high, therefore she was nominated for the Balloon Kyphoplasty as an interventional procedure.

**Conclusions:**

After Kyphoplasty, her pain was immediately resolved, vertebral body height was restored to normal, and pain score was reduced from 8 to 1. She was discharged with a normal neurological examination and good general condition, and returned to her normal life. In this case, Balloon Kyphoplasty resulted in the restoration of the vertebral body height, decrease in pain, and returning to daily activity. Therefore, this technique can be an appropriate alternative for surgery in cardio-pulmonary compromised patients.

## 1. Introduction

Prevalence of compression fractures increases by age and can be primary or secondary because of osteoporosis or malignancy. Vertebrae compression fracture due to osteoporosis is a common problem with a prevalence of 1/4 million people in the world (1). 25% of women in menopause go through this kind of fracture (2). 30% of fractures happen during sleeping, but in moderate osteoporosis, a stronger force like falling down is needed (3). This kind of fracture is very painful and limits the patient’s movements which can lead to secondary osteoporosis. Immobility can increase the risk of pulmonary and extremities emboli. Secondary biomechanical effects of kyphosis in vertebral fractures, increase the risk of re-fracturing and decrease pulmonary capacity and overall, decrease the patient’s life time (4). Primary signs of compression fracture of vertebrae last 4–6 weeks. In some cases, even with long time treatments, sever and resistant pain, would not be resolved, but pain management is essential in these patients (5). Vertebral compression fractures do not response very well to medical treatments and in the most cases, surgery is not a choice because most of these patients are not in a good physical condition. Surgery is mostly done when there is an anatomical compression of vertebral column with luxation of bone and nerve complications. On the other hand, when there are multiple vertebral fractures, performing surgery has a lot of limitations (6). Recently, interventional techniques for augmentation of the vertebral column, has provided less invasive methods. Today Vertebroplasty and Kyphoplasty are known as the replacement methods for surgery in non-responding cases to conservative treatments or sever and resistance pain. The point of these report, is to show the effect of Kyphoplasty in decreasing a patient’s pain that although the patient is suffering from severe pain, but because of symptoms and sever cardiovascular disease, surgical procedure is not an acceptable approach.

## 2. Case Presentation

**Figure 1:**
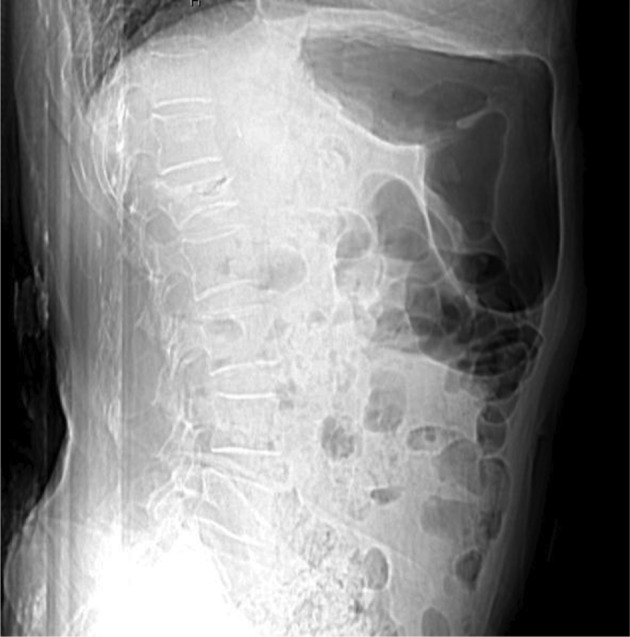
Lateral View of Lumbar Vertebral Column With L1 Vertebral Compression Fracture

**Figure 2:**
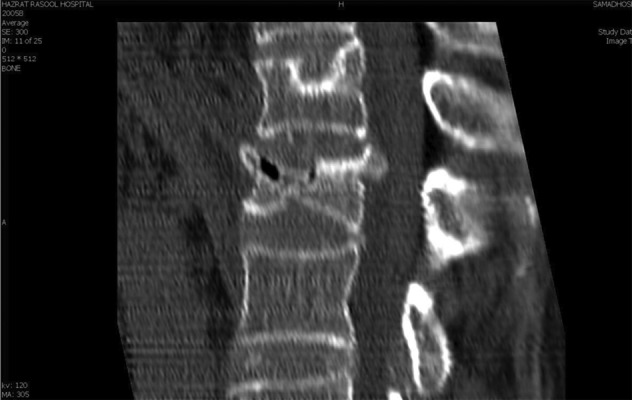
CT Scan of Lumbar Vertebral Column With L1 Vertebral Compression Fracture

**Figure 3:**
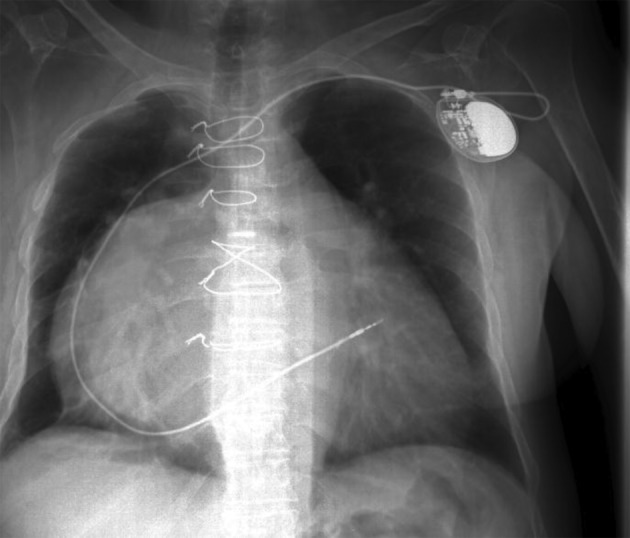
Chest Radiography

A 65 year old woman was referred with the compression fracture of the first lumbar vertebrae (L1) due to falling down, two week ago. Her visual analogue pain score (VAS) was 8 out of 10 in lumbar region, and did not had paresthesia or limb weakness. CT and MRI showed acute wedge fracture of the first lumbar vertebrae which resulted in 30%–40% decrease of vertebral height *(Figure 1 and 2)*. In the physical examination, no nerve defect and intestine or urinary bladder dysfunction were seen. In straight leg raising test (SLR), no radiculopathy sign was seen. In palpation, first to forth lumbar vertebrae were painful. The patient had an acute heart diseases (33%) ejection fraction, heart’s enlargement, twice its standard size and pace maker. The patient was diabetic and had high blood pressure and pulmonary disease (COPD) *(Figure 3)*. The patient was taking 15 drugs daily (such as dilantin, losartan, spironolacton, furosemide, warfarin, digoxin, citalopram, serteralin, alprazolam, clonazepam, atrovastatin, aspirin, salmetrol, flixotide, and carvedilol). Laboratory tests were normal except coagulation time due to the intake of warfarin for cardiovascular problems and after correcting that she was a candidate for Kyphoplasty.

**Figure 4:**
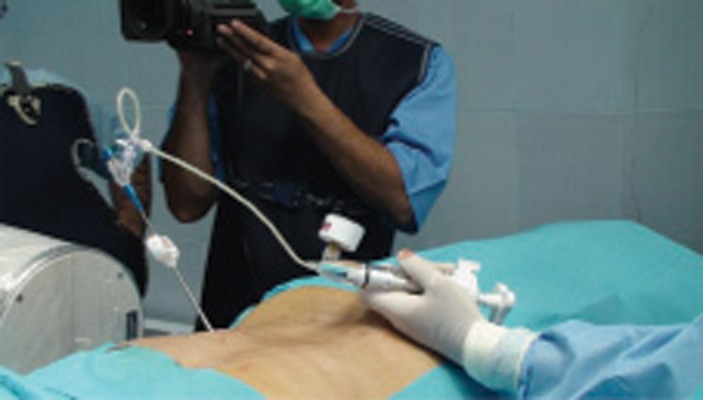
Balloon Inflation and Contrast Injection

**Figure 5:**
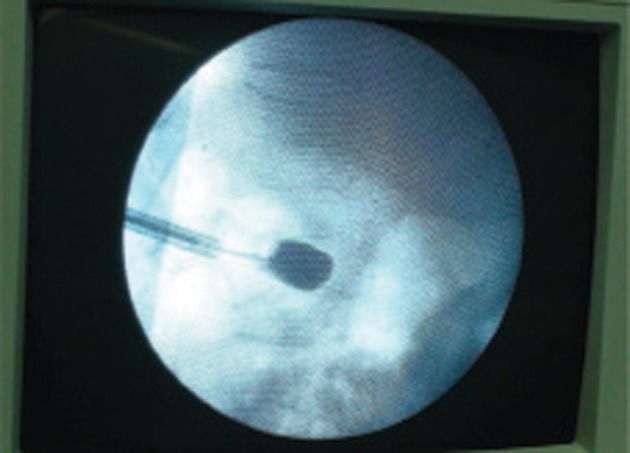
Fluoroscopic View of Balloon Filled by Contrast Media

**Figure 6:**
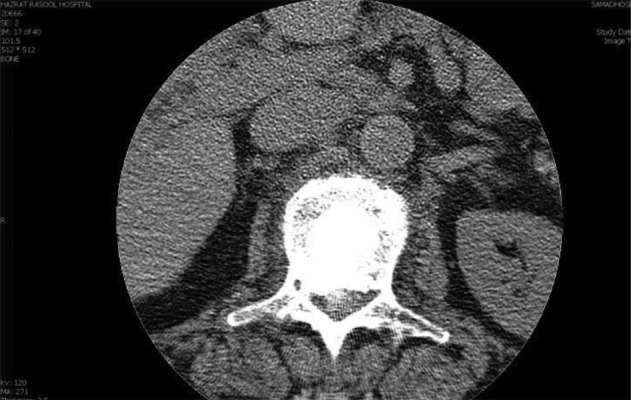
CT Scan After Kyphoplasty (Vertebra is Filled With Cement)

After i.v. premedication (fentanyl 50 mcg and midazolam 1 mg), cefazolin 1 g and hydrocortisone 100 mg were injected. Under sterile condition, an anterior-posterior projection was obtained by fluoroscopy, so that the first lumbar and its pedicle were detected. Then, the fluoroscope was moved 10 degrees towards caudo-cephalic direction and 20 degrees oblique so that the pedicle was seen as a circular shape. The entrance of the needle was chosen in 1/4 outer-upper of the pedicle by Transpedicular approach. Local anesthesia was made by lidocaine 1%. A 5 mm skin incision was done. The trocar needle was introduced by fluoroscopic guidance in a tunnel view and the needle was advanced. After confirmation of the correct position of the needle in the several lateral and anteroposterior views in the vertebral body, the guide wire was placed inside of the needle, and the first trocar was replaced by No.11 trocar over the guide wire. After that, the guide wire was removed, and a drill was placed into the trocar. By drilling, a primary cavity was made in the vertebral body to provide for insertion of the balloon into the body. Then, the drill was removed, and the containing balloon with contrast media was placed into the cavity and was inflated up to 15 PSA *(Figure 4 and 5)*. The balloon was kept in this position for 5 minute restore the vertebral height. Then two filler needles were filled up by 2 ml of cement (Poly-Methylene-Methacrylate). The filler needle was replaced instead of the balloon, and 4 ml of cement was injected under the live fluoroscopy, in order to control the leakage of cement into the spinal cord and disc. The patient was kept in the same position for 15 minutes for cement stabilization. The patient was monitored in the recovery. After two hours, a CT scan was taken, and the vertebral body was correctly filled by cement and no evidence of leakage was obsereved *(Figure 6)*.

After the procedure, the pain was decreased quickly and the pain score (VAS) was decreased to 1. The patient was discharged next day with overall good condition and without any neurological complications. During two next weeks, she was in a very good condition.

## 3. Discussion

Kyphoplasty is one of the strengthening methods of the vertebral column which is used to stabilize the compression fracture and the height of the vertebrae with injecting the cement (Poly-Methylene-Methacrylate) through a needle inside the vertebral body. The analgesic mechanism of cement are including: 1, Analgesic effects of cement; 2, Fixation mechanism of fractured vertebrae; 3, Countering with the osteoclastic activity which is the reason of neuropathic pain due to sensitive vanilloid receptors and ionic channels to acid by changing the extra-cellular acidic environments (7), and 4, Thermal neurolysis of affective bone nerves by polymerization of Poly-Methylene-Methacrylate which forms at 70 degree heat, (8). Also, vertebral body augmentation methods were applied for managing the collapsed or painful vertebrae due to the hemangioma, metastasis, osteoporosis, bone lesion, and myeloma (9). The most important indication of this technique is in the painful compression fracture of thoraco-lumbar vertebral column. If the compression fracture was observed in the imaging study, it’s necessary to distinguish between an acute painful vertebral fracture and chronic pains with a history of old fracture. MRI is helpful for diagnosis of the age of vertebral deformity and patients with a chronic pain due to vertebral fractures who have new evidence of fracture in MRI are good candidates for Kyphoplasty (10).

Vertebral augmentation methods are including Vertebroplasty and Balloon Kyphoplasty. Vertebroplasty is effective in decreasing the vertebral compression fracture pain, but since cannot correct the kyphotic vertebral body deformity, the bio-mechanism of normal vertebral column might be affected. Therefore, it is always a question whther it is a logical approach to fix a vertebral compression fracture without decreasing its deformity. There is also a concern in the process of injecting the cement. Injecting Poly-Methylene-Methacrylate with low viscosity into the vertebrae and fixing the fracture vertebrae increase the risk of leakage and pressure on spinal cord and nerve roots and the risk of nerve dysfunctions.

In Kyphoplasty a balloon enters into the fractured vertebrae and then it blows up and creates a cavity, which will be filled up with the cement. In this method, vertebral height is usually well restored. The indications of Kyphoplasty and Vertebroplasty are similar, but with lower risk of cement leakage, because the cement used in Kyphoplasty is more viscose and, needs less force to be injected into the body (11). In addition, cavity formation by Kyphoplasty decreases the need of forceful injection and risk of leakage.

After Kyphoplasty, the back pain is usually dropped immediately. Its success in decreasing pain is 75%–90% (12). The pain was less decreased in cancer patients than in osteoporotic ones which are likely due to variety of parameters in creating pain in these patients. The maintenance of pain relief in Vertebroplasty was seen from 2 to 5 years (8).

Kyphoplasty can be suitable for restoring vertebral body height and decreasing pain after compression fracture in compromised cardiovascular patients, who did not a good candidate for surgery, without any complications.
